# Supplementary figures and images for: Molecular characterization of the conoid complex in Toxoplasma reveals its conservation in all apicomplexans, including Plasmodium species
Source: PLoS Biol. 2021 Mar 11;19(3):e3001081. doi: 10.1371/journal.pbio.3001081 (PMC7951837; doi:10.1371/journal.pbio.3001081)

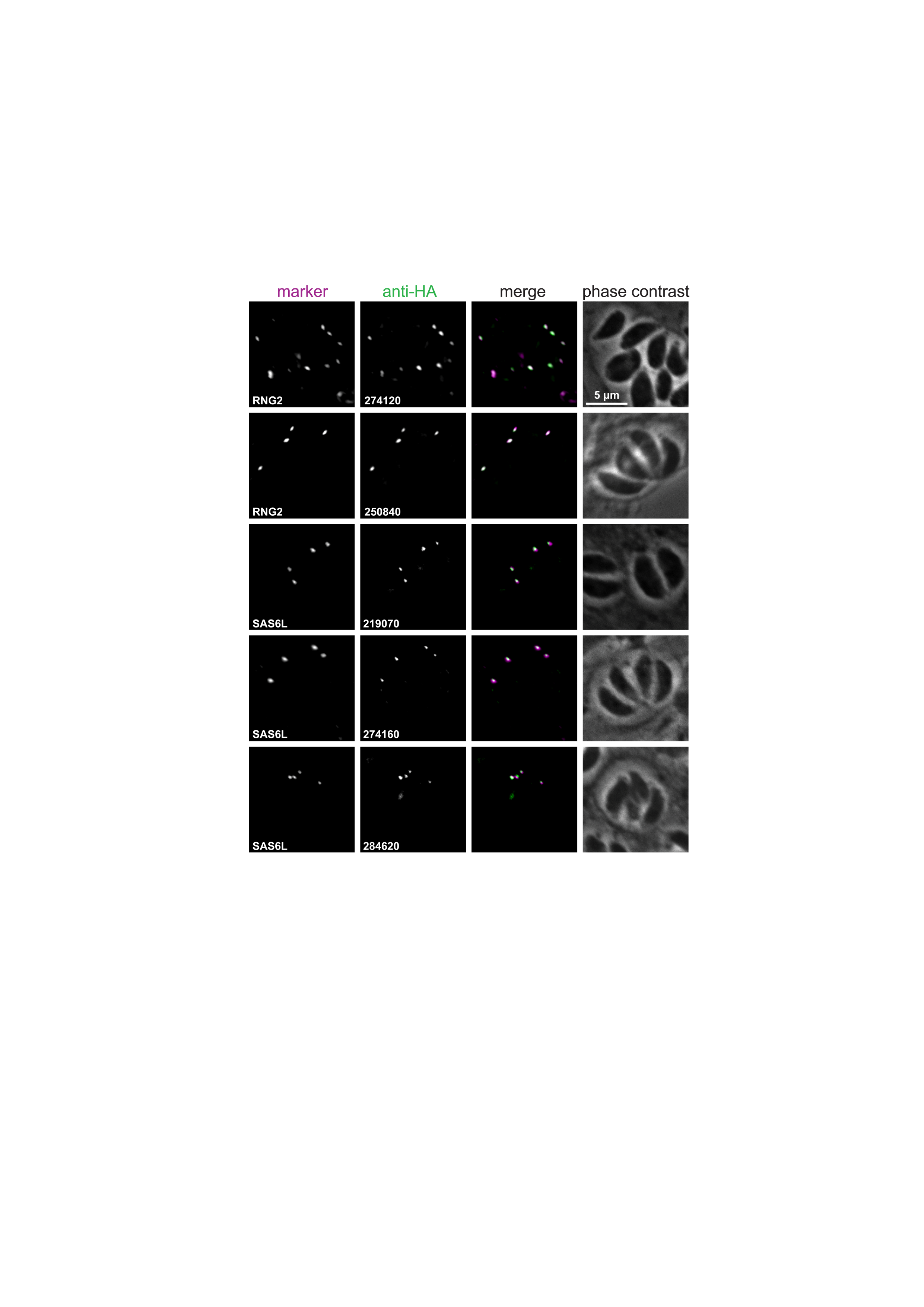

Supplement: S1 Fig — Widefield fluorescence imaging of HA-tagged candidates (green) coexpressing either APR marker RNG2 or conoid marker SAS6L (magenta). All images are at the same scale, scale bar = 5 μm. APR, apical polar ring; BioID, proximity-dependent biotin identification; HA, hemagglutinin; hyperLOPIT, hyperplexed Localisation of Organelle Proteins by Isotope Tagging; SAS6L, SAS6-like. (TIFF) [file pbio.3001081.s007.tiff]

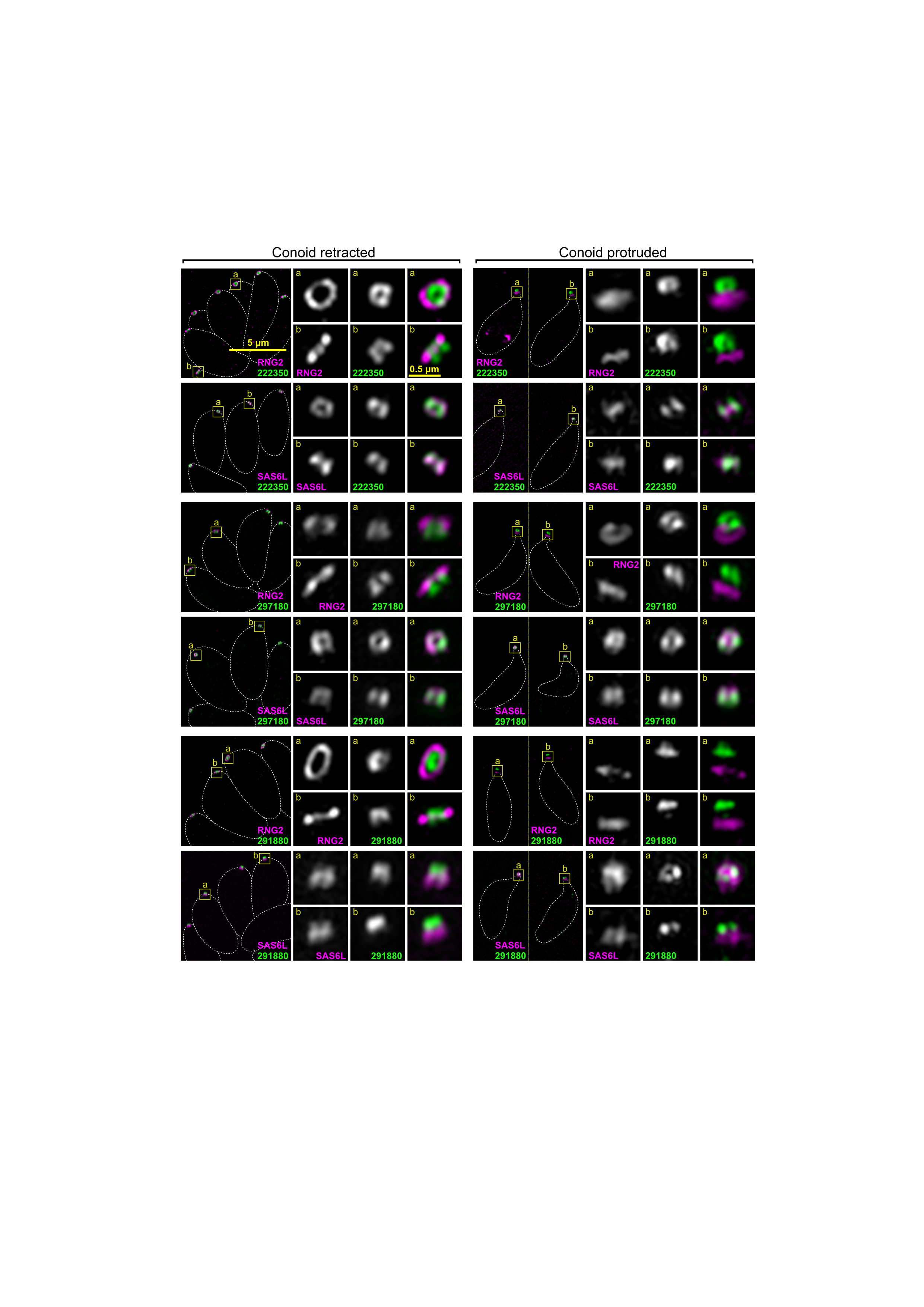

Supplement: S2 Fig — Immunodetection of HA-tagged conoid proteins (green) in cells coexpressing either APR marker RNG2 or conoid marker SAS6L (magenta) imaged either with conoids retracted within the host cell, or with conoids protruded in extracellular parasites. This figure shows further examples of conoid body proteins to those shown in Fig 3A. All panels are at the same scale, scale bar = 5 μm, with zoomed inset from yellow boxes (scale bar = 0.5 μm inset). APR, apical polar ring; HA, hemagglutinin; SAS6L, SAS6-like. (TIFF) [file pbio.3001081.s008.tiff]

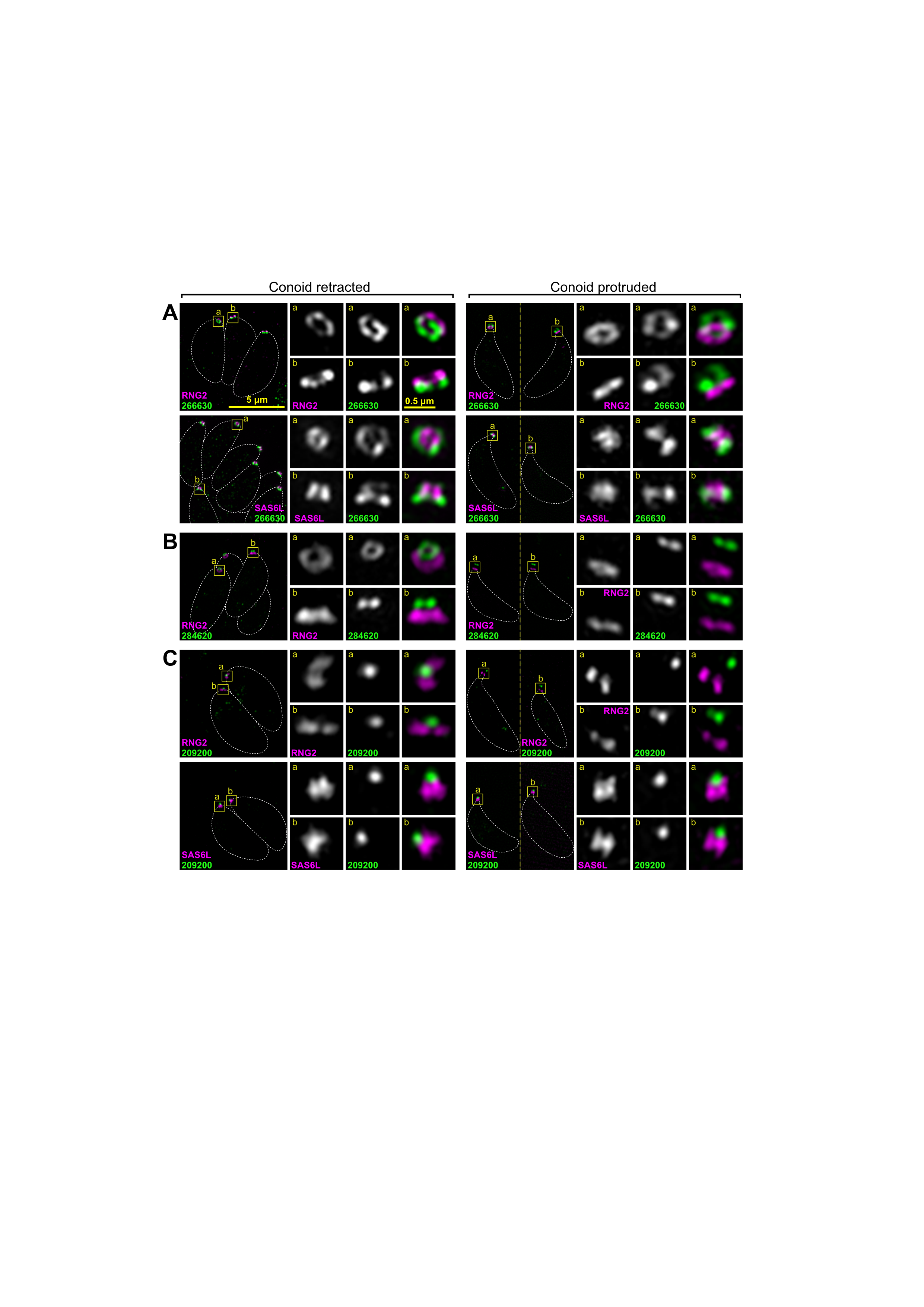

Supplement: S3 Fig — Immunodetection of HA-tagged conoid proteins (green) in cells coexpressing either APR marker RNG2 or conoid marker SAS6L (magenta) imaged either with conoids retracted within the host cell, or with conoids protruded in extracellular parasites. This figure shows further examples of conoid proteins to those shown in Figs 3B, 4A and 5A. All panels are at the same scale, scale bar = 5 μm, with zoomed inset from yellow boxes (scale bar = 0.5 μm inset). APR, apical polar ring; HA, hemagglutinin; SAS6L, SAS6-like. (TIFF) [file pbio.3001081.s009.tiff]

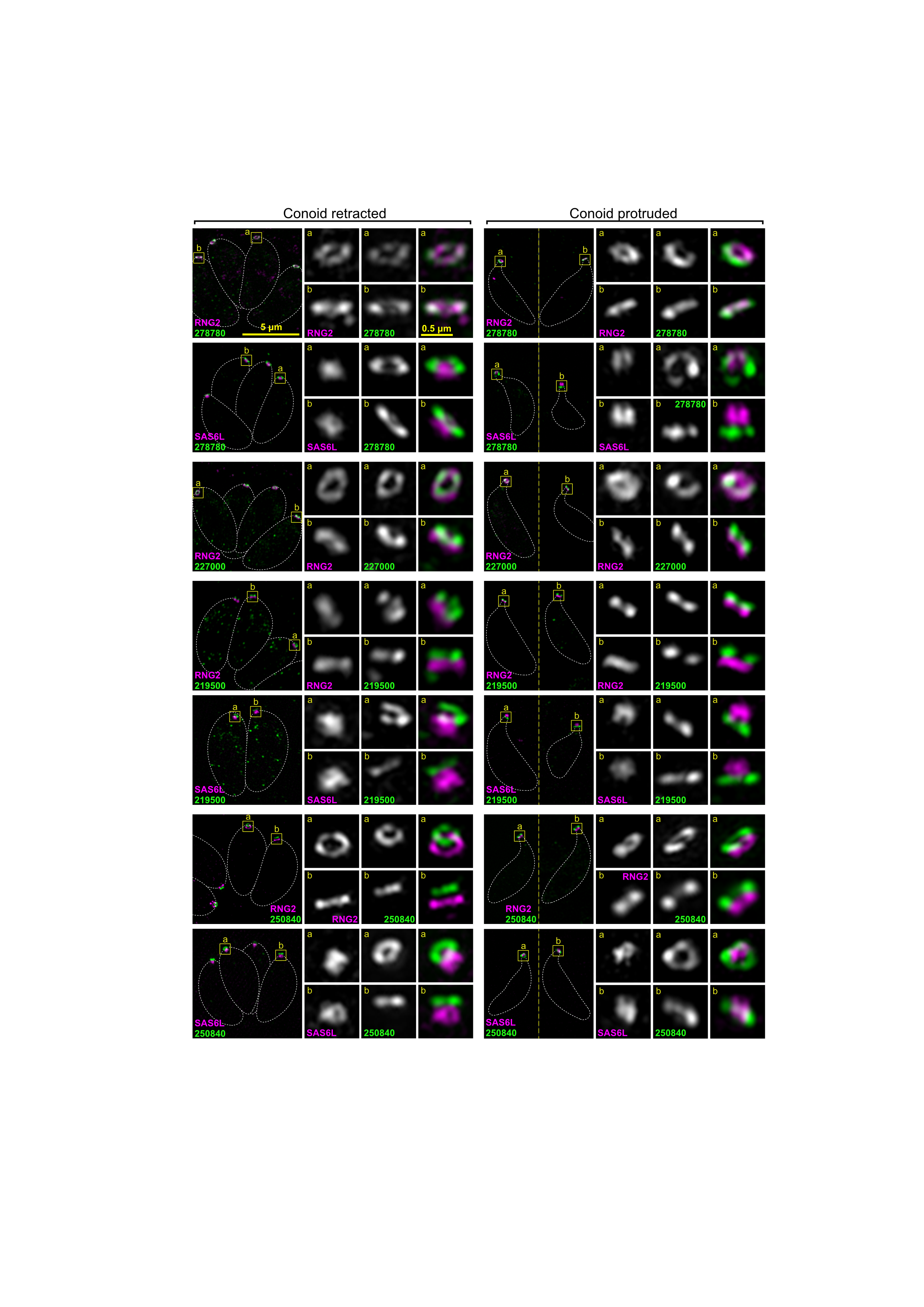

Supplement: S4 Fig — Immunodetection of HA-tagged proteins (green) in cells coexpressing either APR marker RNG2 or conoid marker SAS6L (magenta) imaged either with conoids retracted within the host cell, or with conoids protruded in extracellular parasites. This figure shows further examples of apical polar ring proteins to those shown in Fig 5B. All panels are at the same scale, scale bar = 5 μm, with zoomed inset from yellow boxes (scale bar = 0.5 μm inset). APR, apical polar ring; HA, hemagglutinin; SAS6L, SAS6-like. (TIFF) [file pbio.3001081.s010.tiff]

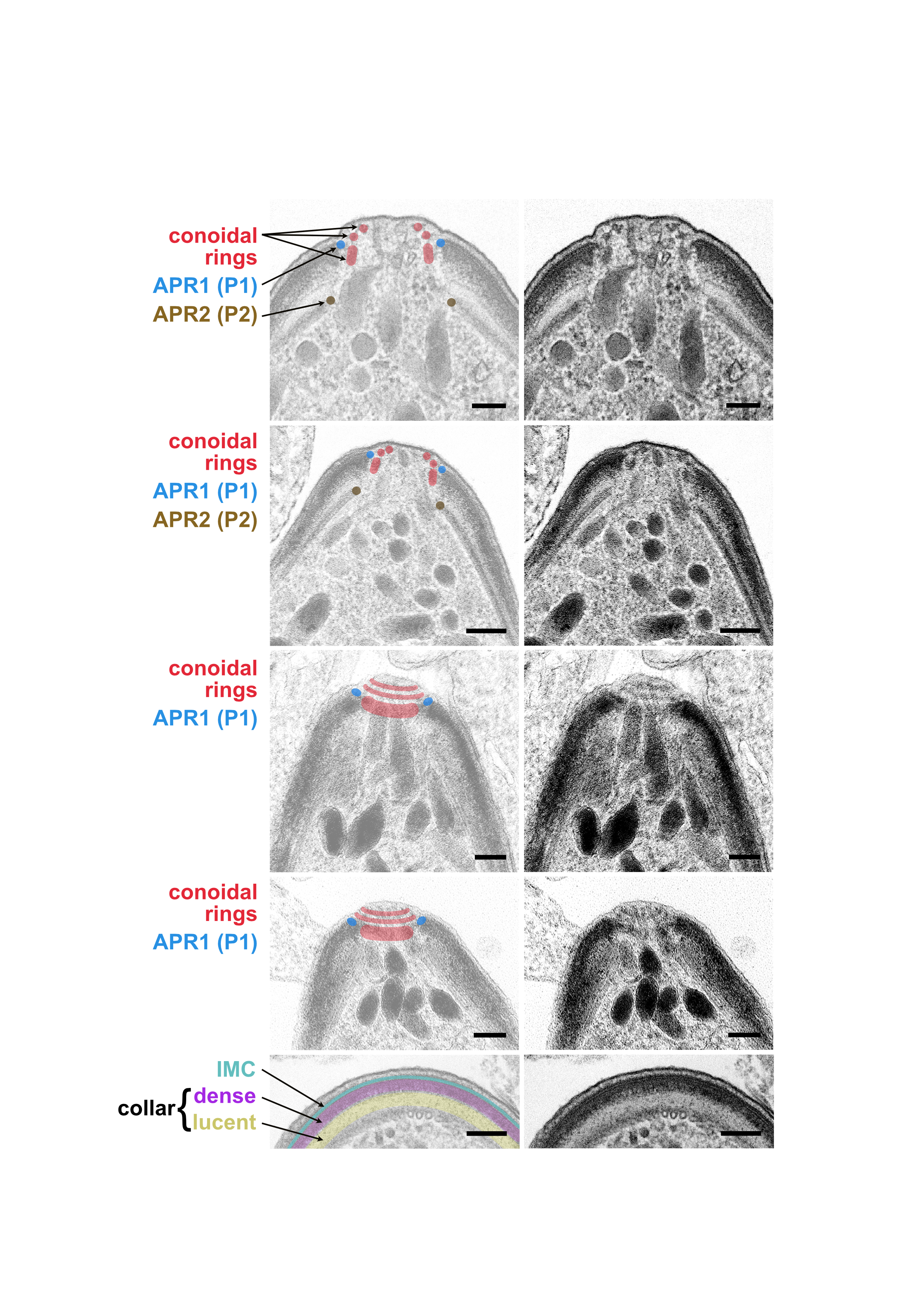

Supplement: S6 Fig — Transmission electron micrographs of P. berghei ookinetes taken from Fig 8 with conoid complex features annotated in image duplicate. Scalebar = 100 nm. APR1, apical polar ring 1; APR2, apical polar ring 2; IMC, inner membrane complex. (TIFF) [file pbio.3001081.s012.tiff]

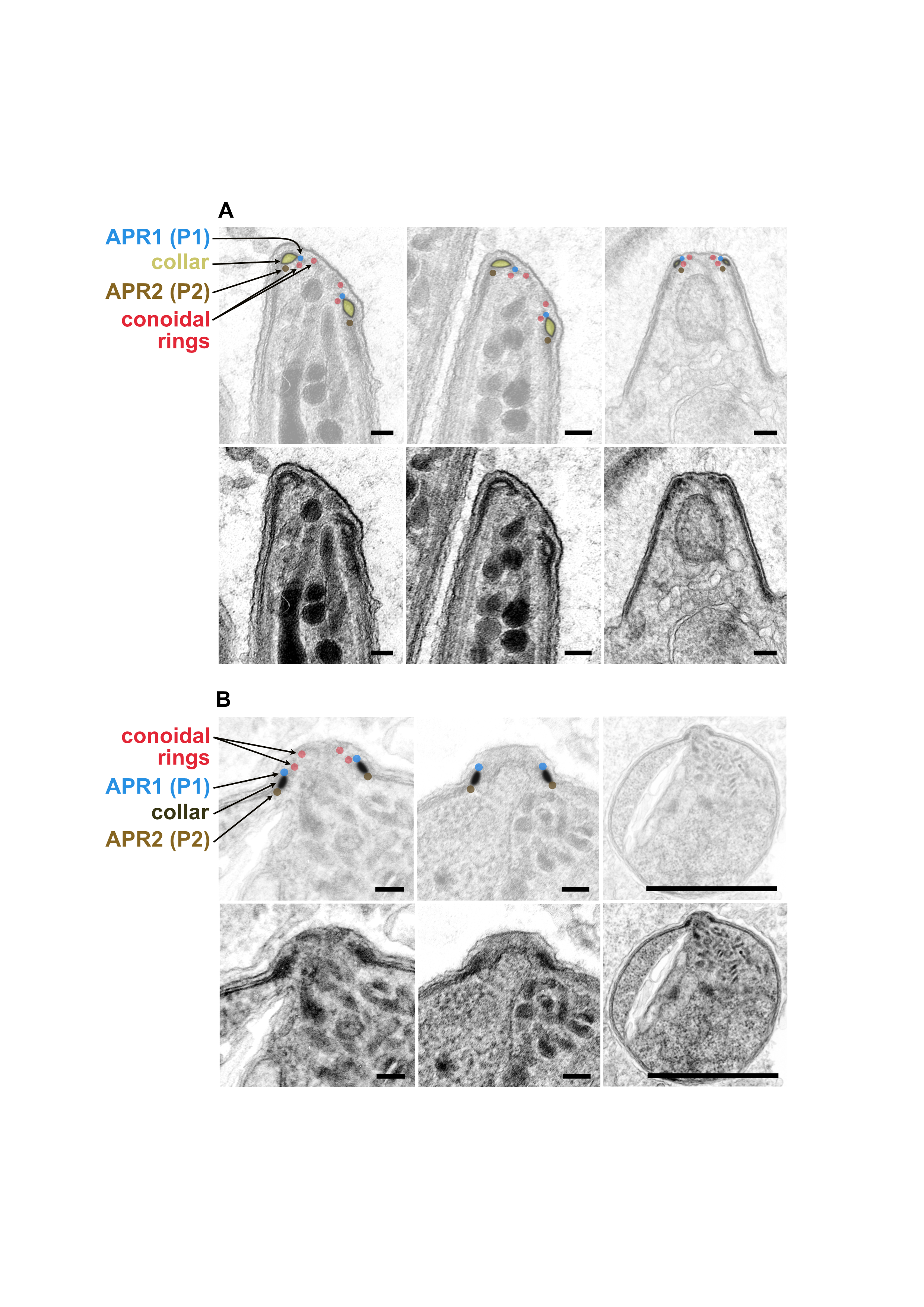

Supplement: S7 Fig — Transmission electron micrographs of P. berghei A. sporozoites and B. merozoites taken from Fig 8 with conoid complex features annotated in image duplicate. Scalebar = 100 nm for all except righthand panels of B. where scalebar = 1 μm. APR1, apical polar ring 1; APR2, apical polar ring 2. (TIFF) [file pbio.3001081.s013.tiff]

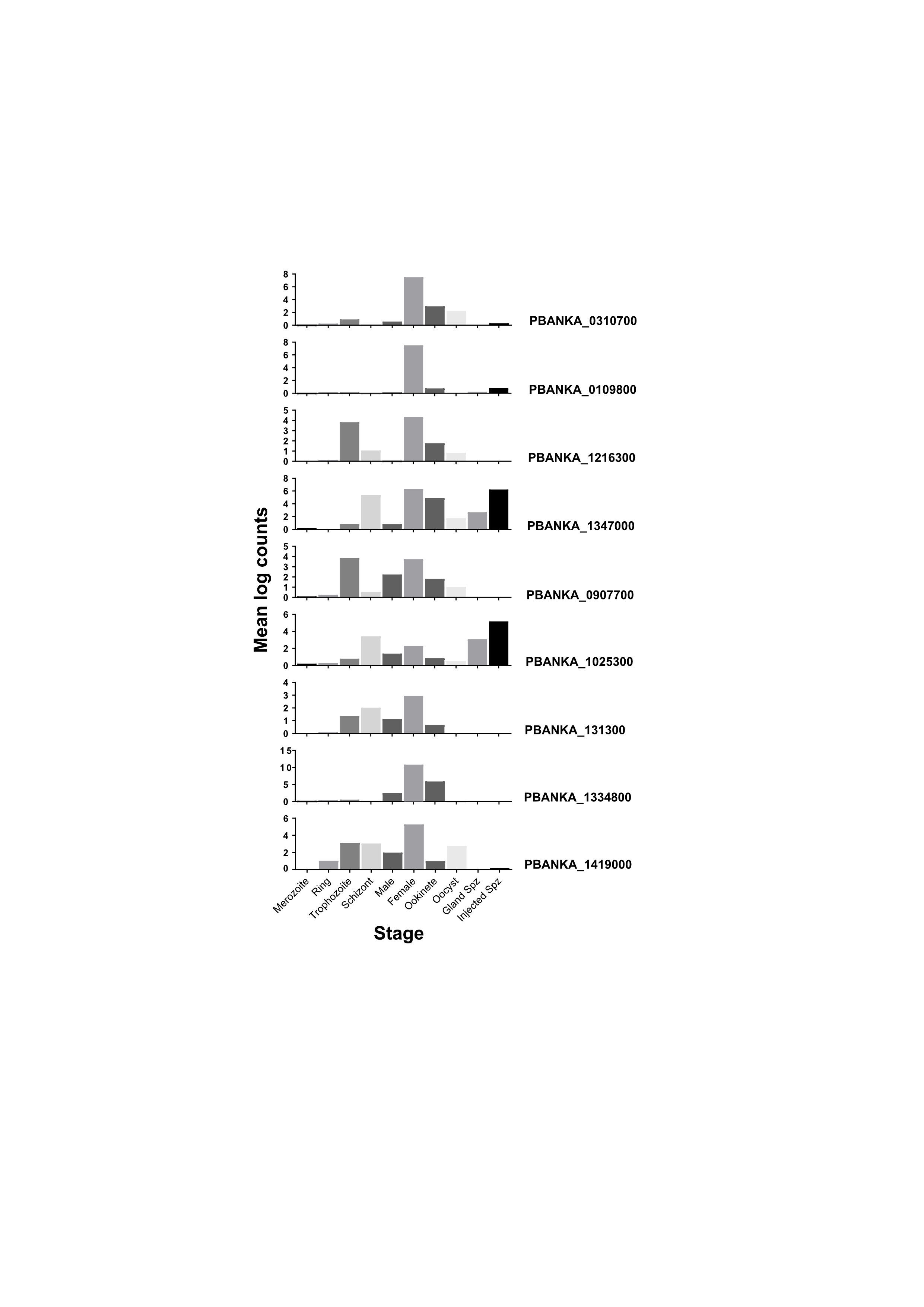

Supplement: S8 Fig — Mean normalised log counts of transcripts for each gene in different stages of Plasmodium berghei from single-cell RNAseq data [81]. See S2 Data for numeric values taken from PLasmoDB. (TIFF) [file pbio.3001081.s014.tiff]

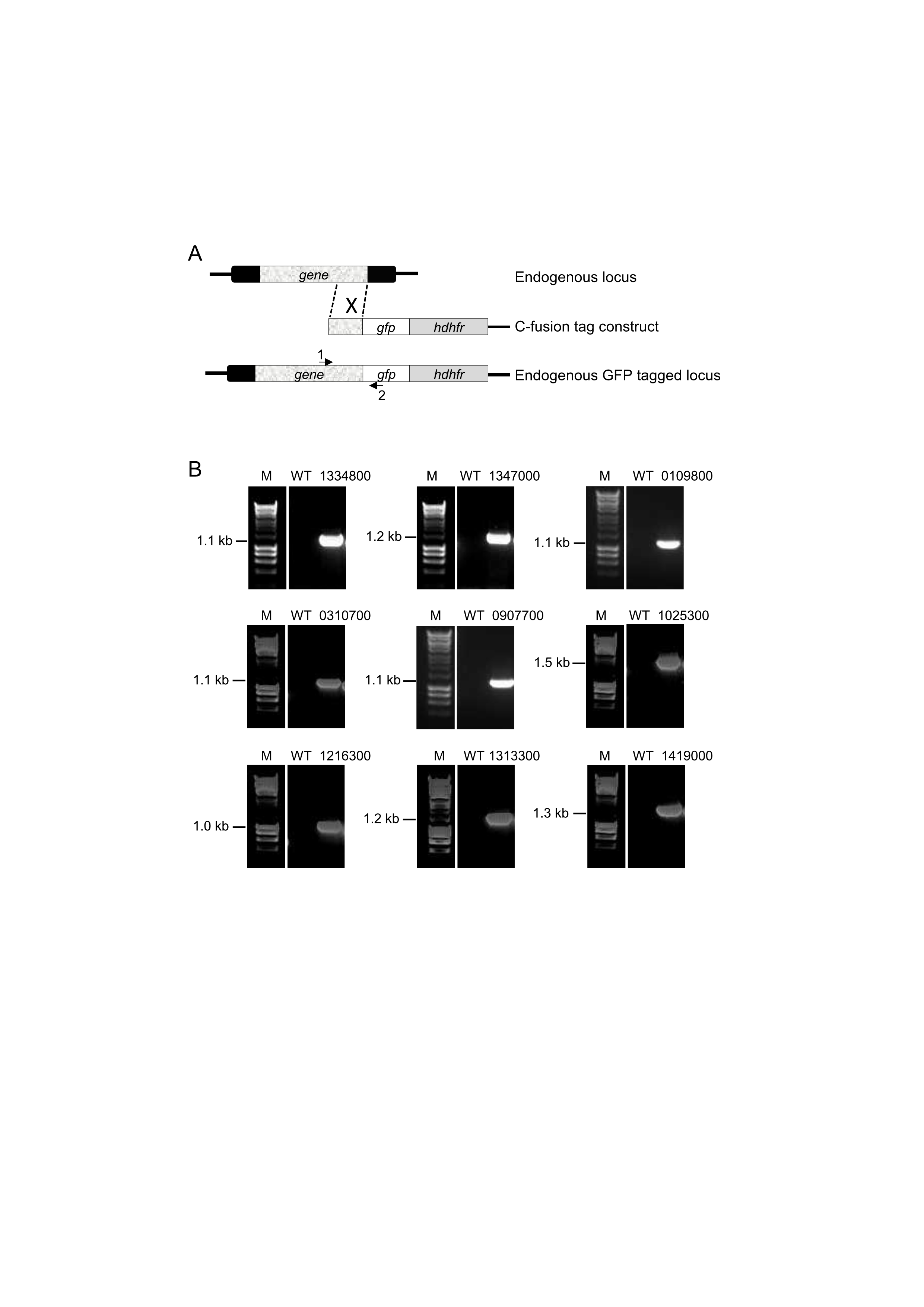

Supplement: S9 Fig — (A) Schematic representation of the P. berghei endogenous gene locus, the integration constructs, and the recombined gene locus. (B) Validation of correct gene tagging by diagnostic PCR. (TIFF) [file pbio.3001081.s015.tiff]
