# Supplementary material for: Molecular characterization of the conoid complex in Toxoplasma reveals its conservation in all apicomplexans, including Plasmodium species
Source: PLoS Biol. 2021 Mar 11;19(3):e3001081. doi: 10.1371/journal.pbio.3001081 (PMC7951837; doi:10.1371/journal.pbio.3001081)
Supplement: Attachment — Submitted filename: Response to Reviewers.pdf [file pbio.3001081.s017.pdf]

We are grateful for the insightful, constructive and very positive reviews provide by the three reviewers below. Accordingly, we present our responses, description of our revisions, and a description of final data and analyses to be included that we have now near completed. This is summarised below and with individual responses to each of the reviewers' comments.

Based on suggested changes to the text we have provided a revised version of the report. All changes made are detailed after the reviewer's corresponding comment.

Further, in response to reviewer suggestions, all *Toxoplasma* conoid-associated proteins reported in this study have now been expressed with both apical markers (SAS6L for the conoid body, RNG2 for the apical polar ring 2) to provide further insight to the new conoid proteins' locations and behaviours. To do this we have generated approximately 20 new transgenic cell lines, and imaging and analysis of these cell lines is nearly complete. Additionally, we are imaging these cell lines with the mobile conoid in its two positions—retracted and protruded. This provides further insight into what structures each protein is attached to. These new data provide significant further detail to the architecture of the conoid complex.

Furthermore, we have extended our analysis of conoid complex orthologue presence/absence in related relevant taxa. We have obtained access to genomic data for a fuller range of apicomplexan and other related myzozoan taxa and uniformly applied our search strategy using Hidden Markov Model approaches. The overall outcome of this extended analysis is unchanged from our initial report, however, it is more complete with respect to taxa relevant to the apical structures of this study. This will extend the relevance of our study to further organism groups, and an even wider field of biologists.

Please find our individual responses to each of the reviewer comments below.

Reviewer #2 (Evidence, reproducibility and clarity (Required)):

The authors study proteins localised to the apical end of the highly polarised parasites causing Toxoplasmosis and malaria. They find new proteins using BioID and examine the localisation of these along with recently identified proteins in the two different parasites. They key question they address is whether there is a conservation of the apical components in these distantly related parasites as well as in some even more distantly related organisms. This is an important question as the apical part comprises many essential proteins of invasion of host cells and shows a unique structure that defines the apicomplexans as a group. The apical structure can be highly elaborate such as in T. gondii and less elaborate as in P. falciparum. The authors now show that there is a large conservation between the species in the protein makeup of the apical end. The experiments are well performed, displayed and discussed and there is no doubt about the validity of the presented results. The text is eloquently written, if at times a bit wordy.

My only main suggestion would be to possibly add data on gene disruption of the two candidates (0310700 and 1216300) that are not detected in blood stage parasites but in the insect stages. A deletion of these should be technically straightforward and would show whether the proteins are important to the parasite. Likely not all of the now many proteins are essential for the parasites but these are good candidates to rapidly investigate. But showing a functional impact might convince editors at certain journals.

Authors' response: The central aim of this study was to ask if the molecular composition of the conoid complex is conserved across Apicomplexa. Functional dissection of proteins is part of an exciting set of subsequent questions and studies that will now follow by us and others. However, careful and thorough phenotyping of gene disruptions is not trivial work, would be most informative to perform in both Toxoplasma and Plasmodium, and is therefore beyond the scope of this project. Regarding the two proteins suggested by this reviewer for follow-up work and the question of 'essentiality', given that these proteins have not been lost during parasite selection through evolution, this is clear evidence of their relevance to the biology of Plasmodium.

Other suggestions in chronological order (line numbers would have helped)

title: maybe write 'conoid complex proteome'

Authors' response: while we initially thought that this change would be suitable, given that the subsequent part of the title is 'reveals a cryptic conoid feature' we think it is clearer and more logical to leave this title in its original form. The conoid complex includes the apical polar rings, and these are not considered to be cryptic or previously unrecognised, only the conoid. While our study confirms that there is conservation across all proteome components of the conoid complex, this is secondary to the primary question of this study.

abstract: not sure about the use of the words instrument and substructures

Authors' response: we believe that the use of 'instrument' is an appropriate analogy of a tool and not different from the use of 'machine' and 'machinery' that is widely used in molecular and cellular biology. Similarly, 'substructure' acknowledges that within recognised structures, such as the conoid, there is further specific organisation such as the conoid base or apex.

page 2 last lines: is tubulin monomeric or polymerized?

Authors' response: to specify the polymerized state of tubulin as mentioned here the text has been changed to 'the presence of tubulin polymers'.

page 3 name protein talked about in 9th line

Authors' response: we have now named this protein (RNG2) as suggested.

third paragraph: mention previous proteomics studies e.g. from Ke Hu (mentioned later in discussion)

Authors' response: We feel that it is more appropriate to leave the discussion of the Hu et al (2006) proteomics study, along with various subsequent approaches used in pursuit of discovering conoid-associated proteins, to the discussion as currently occurs. In the introduction we seek to efficiently inform the reader of the current state of knowledge that makes the value and nature of the questions that we have asked in this study apparent. But we do give full credit and evaluation of previous studies in the discussion which we think is the most appropriate place for this.

first paragraph or results could go into introduction

Authors' response: The first paragraph of the Results contains specific detail of just one aspect of this study, the use of hyperLOPIT. This is relevant to the new analysis that we have made of the hyperLOPIT data in this study. We, therefore, believe that it is most appropriately presented here in the Results in association with the new analyses we described. Our aim is that the Introduction is succinct and serves the entire study.

page 4: add reference after BioID

Authors' response: reference added as suggested

page 5: add definitions of the conoid; what technique was used to report YFP-SAS6?

Authors' response: It is unclear what this reviewer is requesting with respect to definitions of the conoid on this page. Nevertheless, we have now included a thorough definition of the conoid based on the original electron microscopy studies (fourth paragraph of the Introduction).

With respect to the technique used to report on YFP-tagged SAS6 in the de Leon et al 2013 study, we now include fuller description of this previous study as follows:

> *'The fluorescence imaging used in the de Leon et al study was limited to lower resolution widefield microscopy. Immuno-TEM was also used, however, contrary to their conclusions, did show YFP presence throughout transverse and oblique sections of the conoid consistent with our detection of SAS6L throughout the conoid body.'*

page 7: 'showed similar localisation' instead of 'phenocopied'?; add reference after ookinete stage; add expression levels from PlasmoDB to the Table 1 data at least for merozoites, ookinetes and sporozoites or add separate table for the 9 proteins in supplement

Authors' response: 'phenocopied' replaced, as suggested. Reference added after ookinete stage, as suggested.

As requested, we have complied available expression data for the Plasmodium proteins throughout the different zoite stages and will include these data as supplemental material in our subsequent revision.

Discussion:
Maybe discuss that the conoid complex is a cytoskeletal structure and that the other cytoskeletons (actin, microtubules, subpellicular network) also differ between the species investigated in their composition and overall architecture

Authors' response: These are reasonable suggested analogies and we can introduce them in the subsequent revision.

page 9: at least two proteins could be deleted as they seem to not confer any growth defect on blood stages (see main comment)

Authors' response: This reviewer has not linked this comment to a specific statement on page 9, however, we are cautious not to interpret lack of observed growth defects in experimental scenarios with unimportant or irrelevant proteins. Maintenance, through natural selection and evolution, of proteins of a structure indicate that they are selectively advantageous and of functional relevance. The two proteins in question are not expressed in the blood stage, so one wouldn't expect their deletion to have consequence in this stage.

Apart from classic TEM images also Cryo EM data is available for apex of merozoite and sporozoite. Worth to discuss?

Authors' response: According to this review's subsequent suggestion (below), we have included a schematic of the zoite stages of Plasmodium and these draw on Cryo EM tomography data.

Add and discuss the recent work from Curr Biol and EMBO J of the Yuan lab on ookinete formation?

Authors' response: These two reports are excellent studies of the polarised development of the cell pellicle during ookinete formation and control of gliding initiation, but don't specifically related to the conoid complex structures that are the subject of our study. We, therefore, do not see a logical place to include discussion of these works.

Reviewer #2 (Significance (Required)):

The paper provides a conceptual advance over previous data as it shows clearly a high level of conservation of the protein components of the conoid complex. It could introduce a new

terminology for these important apical structure of Apicomplexan parasites and provides a good basis to dissect the molecular functions.

Authors' response: We appreciate this reviewer recognising this opportune point in time to more clearly define the terminology applied to these apical structures so that they can be more clearly and easily compared between taxa. This has now been done in the revision along with clear schematics that show the equivalent structures between taxa and zoite forms. This also makes this important aspect of apicomplexan biology more accessible to general readership. We have used terminology based on precedent in the original ultrastructural literature.

As it stands all scientists investigating Plasmodium and Toxoplasma invasion of host cells will be highly interested in this study, most scientists researching apicomplexan organisms should be and some evolutionary scientists will be interested in this study.

Key papers in the field are the discovery of the Toxoplasma conoid as a highly twisted microtubule-like structure (Hu et al., JCB 2002; doi: 10.1083/jcb.200112086) the first description of an apical proteome (Hu et al., PLoS Path 2006; 10.1371/journal.ppat.0020013), the description of a tilted arrangement of the rings in Plasmodium versus Toxoplasma (Kudryashev et al., Cell Microbiol 2012; doi: 10.1111/j.1462-5822.2012.01836.x) and the discovery of apical located proteins that are essential for conoid formation (Tosetti et al., eLife 2020; 10.7554/eLife.56635) to name a few.

If intended for a broader audience, a cartoon of a conoid complex across the different species investigated and discussed here would help for visual guidance highlighting the similarities and differences

Authors' response: This is a good suggestion and we have acted on this in new figures 1 and 10. Furthermore, we now include transmission electron microscopy to support these cartoon and that further define the apical structures of each of the cell stages examined.

Reviewer #3 (Evidence, reproducibility and clarity (Required)):

In this work, Koreny et al. characterized the localization of a new collection of conoid proteins in Toxoplasma gondii as well as in several different stages of Plasmodium berghei. The authors discovered that these proteins are located in several distinct substructures in Plasmodium and are expressed in a stage-specific manner. The data are of high quality, well-organized, and well presented. The paper is well written. The introduction, in particular, was a pleasure to read. This reviewer (Ke Hu) does not have any new experiments to suggest.

However, while the authors present LOPIT+BIOID as a powerful approach to identify conoid proteins, implying that it is more reliable than previously published approaches (see below), the manuscript includes no data to show what the false positive or false negative rate is with the current approach, nor any estimate of how many conoid proteins were missed entirely.

Authors' response: In our validation of putative conoid-associated proteins identified by the hyperLOPIT+BioID approach we reporter-tagged 18 proteins to resolve their cellular location by microscopy. All 18 were verified as being located at the site of the conoid. So, by this measure there were no false positives. The veracity of the hyperLOPIT data was also confirmed across other cell compartments in our report where 62 proteins were reporter-tagged from which there were no false positive assignments of cell location (Barylyuk et al., 2020, Cell Host & Microbe, in press:doi:10.1016/j.chom.2020.09.011, https://www.cell.com/cell-host-microbe/fulltext/S1931-3128(20)30514-X)

Estimating false negatives is more difficult, but we know that these would occur as for any mass spectrometry-based detection technique. However, we have not claimed to have been exhaustive, nor was this required to answer our central question of are there conserved conoid-associated proteins throughout Apicomplexa? To address this question, we required a good sample of proteins, and the methods that we have employed provided this.

Page 7: "Previous identification of conoid complex proteins used methods including subcellular enrichment, correlation of mRNA expression, and proximity tagging (BioID) (Hu et al. 2006; Long, Anthony, et al. 2017; Long, Brown, et al. 2017). Amongst these datasets many components have been identified, although often with a high false positive rate. We have found the hyperLOPIT strategy to be a powerful approach for enriching in proteins specific to the apex of the cell, and BioID has further refined identification of proteins specific to the conoid complex region."

The authors should state whether the candidate proteins were chosen in an unbiased way or not.

Authors' response: Candidate proteins selected for validation by microscopy were not biased for any known likelihood of being associated with the conoid, other than our proteomics data what we were seeking to test. However, we did preference proteins with the following traits, 1) proteins with strong corresponding gene knockout fitness phenotypes from published studies, 2) proteins with some evidence of conserved functional domains, and 3) genes with orthologues found in Plasmodium spp. and other apicomplexans. These traits were chosen with future functional studies in mind where proteins might be more informative of conoid-related functions and relevance in other apicomplexans. All validated proteins, however, were otherwise uncharacterised and, therefore, were not knowingly biased for more likely conoid-association over others discovered by our proteomics approach. We now include the following statement.

> *"All proteins selected for validation were previously uncharacterised and with no a priori reason to be identified as conoid-associated other than our proteomics data."*

If so, how many proteins were localized to the conoid and how many were not?

Authors' response: as stated above, we observed no false positives from the sample of 18 protein locations verified by microscopy.

Related to this, the majority (14 out of 20) of the conoid proteins identified by LOPIT+BIOID in this paper were previously identified as conoid candidate proteins in Hu et al's 2006 paper, based on the number of peptides retrieved from the conoid enriched vs depleted fractions. Those data (see below) have been available from ToxoDB for many years and should be acknowledged.

Accession# - conoid enriched : conoid depleted (from Hu et al. 2006)

222350 - 2:0

274120 - 3:0

291880 - 1:0

301420 - 3:1

246720 - 4:0

258090 - 10:0

266630 - 8:1

208340 - 4:2

253600 - 1:0

306350 - not found

250840 - 1:0

292120 - not found

219070 - not found

274160 - not found

320030 - 7:1

227000 - 10:0

278780 - not found

284620 - not found

295420 - 6:0

297180 - 4:0

Authors' response: Proteomic methods and mass spectrometry have experienced revolutionary advances since this 2006 study was conducted. These include improvements in both sensitivity and quantitation accuracy. The Hu et al 2006 study provided an exciting first step towards conoid protein discovery. However, by their admission, at least 35% of their putative conoid-specific proteins were identifiable as false positives (e.g. ribosomal proteins) and this estimate could not account for the majority of uncharacterised proteins whose potential for false positive attribution to the conoid was untested. From almost 300 proteins, this study only validated four as associated with the conoid. The further proteins listed above were not validated as conoid proteins in the Hu et al study and, therefore, could not be distinguished from the many false positives reported in their work. In our Table 1, we have acknowledged the Hu et al study for the select proteins that they established as conoid proteins in their study.

To further assess the utility of this 2006 conoid-enriched proteome we sorted the Hu et al detected proteins on our full hyperLOPIT assignments (see stacked bar chart below). Of the proteins that were reported by Hu et al as either exclusive to the conoid-enriched fraction (yellow) or enriched by at least 2-fold over the conoid-depleted fraction (green), 15% were assigned to the apical 1 and 2 clusters (representing the relevant compartments to the conoid complex). Thus, according to the hyperLOPIT data these represent the true positives found in this study and 13 of these proteins were independently validated as conoid-associated by us. Cyan, blue and purple bars in apical 1 and 2 assignments indicate false negatives in their data. Most significantly, 85% of the conoid-exclusive and conoid-enriched proteins from Hu et al (2006) were allocated to a non-apical location with 99% probability by hyperLOPIT, and, during our validation of 62 assignments we verified the alternative location of eight of these. False positives, therefore, greatly outnumbered true positives in this earlier dataset. This high rate of false positives in subcellular isolation proteomics is typical of the challenges that this method faces, and this was the rationale for and strength of the alternative hyperLOPIT approach. Given the overall relatively low level of conoid specificity in the earlier work we do not think that there is value in making specific protein-by-protein reference to it. The analysis below is for the Editor's information only however, if deemed appropriate, this could be included as supplemental information also.

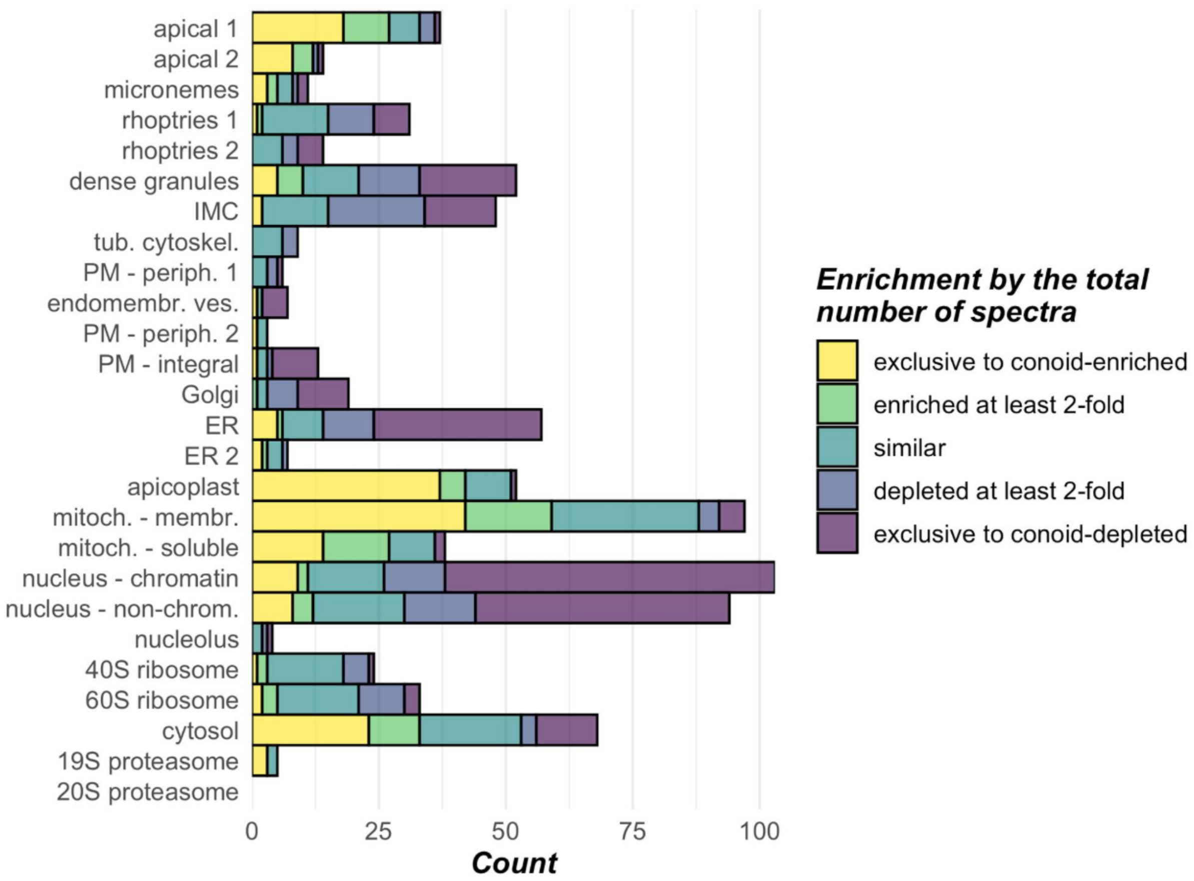

Reviewer #3 (Significance (Required)):

see above

Reviewer #4 (Evidence, reproducibility and clarity (Required)):

This manuscript details the further use of the hyperplexed Localisation of Organelle Proteins by Isotope Tagging (hyperLOPIT) that the group has previous published using T. gondii tachyzoites by combining this with BioID and super-resolution microscopy in order to uncover new proteins that form part of a structurally known and functionally elusive conoid. The authors conclusively identified new proteins that localise to the conoid structure in T. gondii and also excitingly showed that not only is this structure found in all invasive forms of plasmodium (using the P. berghei model) but there also is a different molecular make up in the blood stage merozoites which have a slightly reduced number of proteins (or possible as yet unknown alternatives) compared to ookinetes and sporozoite conoid structures. This study is scientifically sound and the conclusions reached are well supported by the results presented.

Major Comments: No major comments

Minor Comments:

1)While both the introduction and discussion and well written and detailed they could both be a little more concise.

Authors' response: We take this as a style recommendation, but we note that the other reviewers commented on the text's "eloquence" and that the introduction in particular was a "pleasure to read". We take these comments as a votes of confidence in the current form.

2)Selection of the 5 new genes in Tg to be tagged (top pg 5) it was not clear as to the selection criteria for these 5.

Authors' response: Please see the same query, and response with modified text, made by Reviewer #3.

This also leads to the second part of this question where there appears to be some genes missing from Table 1 and Table S1, specifically those found in both SAS6L and RNG2 BioID. It was mentioned that 25 were identified in both SAS6L and RNG2 BioID. In Table 1 (there are 23) there is no mention of 223790, 281650, 224700, and 293540 but they are in the Table S1 (assuming these 4 are not selected in this study for tagging) but in table S1 (there are 25 listed) 216080 (AKMT) and 234250 (CIP1) that are in the Table 1 as being identified in both SAS6L and RNG2 BioID are absent from the Table S1 does this mean there are actually 27 or was the indication of identified in both SAS6L and RNG2 BioID for 216080 (AKMT) and 234250 (CIP1) in Table 1 a mistake?

Authors' response: This reviewer has overlooked that Table 1 reports on all currently known conoid associated proteins, including those not detected in the hyperLOPIT data but reported in the literature, whereas Table S1 is exclusively those proteins detected and assigned as 'apical' by hyperLOPIT. The reported BioID-detection for each protein is then made within this framework. Thus, the proteins that occur in only one or the other table do so because they don't satisfy these two sets of criteria. We have rechecked the numbers reported in the text and they are correct.

3)Table 1: There is the fitness score for Pf orthologues but no mention of fitness in Pb (the model used) from the PlasmoGEM screens, considering the authors use the Pb model it would be of interest to add this in the table.

Authors' response: The Plasmodium berghei PlasmoGEM gene disruption screen were much more limited in number than that for P. falciparum. Consequently, fitness scores were available for only two of the Plasmodium orthologues for which we have location data. We, therefore, thought it was of limited utility to include these data in Table 1, and these data are in the public domain should a reader seek them.

4)Figure 2: The image for localisation with SAS6L for 291880 and 258090 appear to be missing.

Authors' response: Initially we did not make the separate transgenic cell lines for each protein with both the SAS6L and RNG2 markers. This was because one marker was usually sufficient to resolve the relative location of the protein of interest. However, given this reviewer's comment and the potential for some extra information to be recovered by using both markers, we have now generated all cell lines necessary for this analysis. We are presently completing the imaging of these new cell lines and these data will be included in the subsequent revision.

5)Figure 3: It is unclear why both SAS6L and RNG2 are not used for all localisations shown (this could be clarified in the text)

Authors' response: see previous comment.

6)Figure 5: It is a shame only 7 of the 9 plasmodium orthologues were included in the super resolution as there is only 2 more to have the complete set.

Authors' response: Ideally, we would have been able to achieve this but, the restrictions imposed by the COVID-19 disruption to laboratory access and activities ultimately slightly limited these analyses. However, to answer the central question of whether there is conservation of the Toxoplasma conoid proteome in Plasmodium it was not necessary to perform super resolution imaging for all of these proteins. The major outcome of this study, therefore, is not affected by this.

7)Figure 6: As with Figure 5 it would be better if more were included in the super-resolution images in this sporozoite stage.

Authors' response: Same response as above. Generation of sporozoites requires passage through the mosquito vector so this is even more resource-intensive than generation of ookinetes that can be differentiated in vitro from mouse-derived parasites. Again, the answers to the central questions posed by this study do not require these further, high resolution, data.

8)Figure 7: This would be improved with at least a selection (or even all 6) to have the super-resolution images (possibly even with free merozoites)

Authors' response: We did apply 3D-SIM imaging to fixed merozoites, however, unlike ookinetes and sporozoites, the imaged fixed material was inferior to the live cell GFP imaging that we have included. This likely reflects the poorer fixation properties of Plasmodium merozoites that is a challenge of these cell forms that is widely experienced by Plasmodium researchers. We do not have access to a 3D-SIM microscope within a containment laboratory necessary for handling viable parasites, therefore, could not attempt to image live material with this instrument. Again, the answers to the central questions posed by this study do not require these further, high resolution, data

9)As there are numerous new protein identified in 2 different parasites and with the composition of the conoid differing at different stages it would be beneficial to have some sort of schematic model of the apical complex in Tg and Pb indicating where each new protein localises

Authors' response: In response to this reviewer, and reviewer #2's suggestion, we have generated a schematic model (Figure 10).

Reviewer #4 (Significance (Required)):

The authors have combined expert mass spectrometry and super-resolution microscopy to identify new components of the conoid in Tg and added to the knowledge that will help to uncover the function of the structure. But perhaps the most significant is the conclusive identification of the conoid in all 3 invasive stages of the plasmodium parasite. Until now it was widely accepted that the conoid was missing in plasmodium and to uncover multiple proteins that appear to make up and constitute this structure in Plasmodium is highly significant and clear of interest to the Apicomplexean field. Furthermore the suggestion that the conoid differs in the molecular makeup within Plasmodium depending on stage is very intriguing and clearly of interest. This paper expertly combined cutting-edge proteomic and microscopy to identify the conoid in Plasmodium. This manuscript would have a broad readership in parasitology, proteomics, and cell biology

Our expertise is largely in molecular parasitology and microscopy
